# Baseline whole-lung CT features deriving from deep learning and radiomics: prediction of benign and malignant pulmonary ground-glass nodules

**DOI:** 10.3389/fonc.2023.1255007

**Published:** 2023-08-17

**Authors:** Wenjun Huang, Heng Deng, Zhaobin Li, Zhanda Xiong, Taohu Zhou, Yanming Ge, Jing Zhang, Wenbin Jing, Yayuan Geng, Xiang Wang, Wenting Tu, Peng Dong, Shiyuan Liu, Li Fan

**Affiliations:** ^1^ Department of Radiology, Changzheng Hospital, Naval Medical University, Shanghai, China; ^2^ School of Medical Imaging, Weifang Medical University, Weifang, Shandong, China; ^3^ Department of Radiology, The Second People’s hospital of Deyang, Deyang, Sichuan, China; ^4^ School of Medicine, Shanghai University, Shanghai, China; ^5^ Department of Radiation Oncology, Shanghai Jiao Tong University Affiliated Sixth People’s Hospital, Shanghai, China; ^6^ Department of Artificial Intelligence Medical Imaging, Tron Technology, Shanghai, China; ^7^ Medical Imaging Center, Affiliated Hospital of Weifang Medical University, Weifang, Shandong, China; ^8^ Clinical Research Institute, Shukun (Beijing) Technology Co., Ltd., Beijing, China

**Keywords:** ground-glass nodules, lung cancer, deep learning, radiomics, tomography, X-ray computed

## Abstract

**Objective:**

To develop and validate the model for predicting benign and malignant ground-glass nodules (GGNs) based on the whole-lung baseline CT features deriving from deep learning and radiomics.

**Methods:**

This retrospective study included 385 GGNs from 3 hospitals, confirmed by pathology. We used 239 GGNs from Hospital 1 as the training and internal validation set; 115 and 31 GGNs from Hospital 2 and Hospital 3 as the external test sets 1 and 2, respectively. An additional 32 stable GGNs from Hospital 3 with more than five years of follow-up were used as the external test set 3. We evaluated clinical and morphological features of GGNs at baseline chest CT and extracted the whole-lung radiomics features simultaneously. Besides, baseline whole-lung CT image features are further assisted and extracted using the convolutional neural network. We used the back-propagation neural network to construct five prediction models based on different collocations of the features used for training. The area under the receiver operator characteristic curve (AUC) was used to compare the prediction performance among the five models. The Delong test was used to compare the differences in AUC between models pairwise.

**Results:**

The model integrated clinical-morphological features, whole-lung radiomic features, and whole-lung image features (CMRI) performed best among the five models, and achieved the highest AUC in the internal validation set, external test set 1, and external test set 2, which were 0.886 (95% CI: 0.841-0.921), 0.830 (95%CI: 0.749-0.893) and 0.879 (95%CI: 0.712-0.968), respectively. In the above three sets, the differences in AUC between the CMRI model and other models were significant (all *P* < 0.05). Moreover, the accuracy of the CMRI model in the external test set 3 was 96.88%.

**Conclusion:**

The baseline whole-lung CT features were feasible to predict the benign and malignant of GGNs, which is helpful for more refined management of GGNs.

## 1 Introduction

With large-scale lung cancer screening implementation worldwide, more and more ground-glass nodules (GGNs) are detected, and the management pressure is also increasing (1–3). Persistent GGNs on computed tomography (CT) are usually the earliest stage in the development of lung adenocarcinomas (2). For newly detected GGNs, the Fleischner Society, American College of Radiology, and NELSON study gave corresponding management recommendations according to the size and volume of nodules, respectively (4–8). Physicians usually review CT scans after a specific interval (3 months, 6 months, or even one year) to observe the change in GGNs and then decide whether to intervene or continue to follow up according to the growth rate.

However, multiple scans undoubtedly increase the cost of screening and radiation dose on patients. Moreover, anxiety may present throughout the follow-up period and affect life. In addition, the pathological aggressiveness of GGNs may not match the morphological features observed on CT images. For example, some researchers reported that invasive lesions accounted for more than 50% of their cohort of subcentimeter (≤1cm) pure ground-glass nodules (pGGNs), and the traditional conservative treatment recommendations for small pGGNs may miss timely intervention of such lesions (9). Therefore, qualitative diagnosis of GGNs at baseline CT scans, identification of malignant GGNs, and prompt treatment would be beneficial to improve the efficiency of lung cancer screening and reduce the financial and mental burden on patients.

In recent years, many studies have achieved the prediction of benign and malignant pulmonary nodules based on radiomics, and most of them only extract the local radiomics features of the nodules for modeling (10–12). Some studies have also used the information of the surrounding microenvironment of nodules (usually expanding the range of radiomics feature extraction by 2-15mm) for prediction (13, 14). However, there is currently no unified standard for the range of extracted features related to the lung parenchyma around the nodule. Meanwhile, previous studies have proved that features from the whole lung can be used for prognosis prediction or differential diagnosis of local lesions in the lung (15–17). Thus, features that include a more comprehensive range of lung parenchyma may also be used to predict the benign and malignancy of GGNs. Moreover, to ensure the accuracy of lesion segmentation, most current radiomics studies of pulmonary nodules are still carried out by manual or man-machine collaborative semi-automatic methods, which is not only time-consuming and laborious but also subjective factors lead to inter-observer differences in segmentation results (18–20). Inter-observer differences may lead to changes in the extracted radiomics features, affecting the final prediction performance.

With the in-depth development of deep learning (DL) technology in chest imaging, automatic lung segmentation and pulmonary nodule feature extraction can be performed on routine chest CT images (21–23). Besides, to our knowledge, few studies use whole-lung information to predict benign or malignant GGNs. Therefore, to extract the maximum range of lung features and reduce the influence of inter-observer variability, in the present study, we explored the feasibility of using whole-lung baseline CT features deriving from deep learning and radiomics to predict benign and malignant GGNs.

## 2 Materials and methods

### 2.1 Patient inclusion and allocation

The GGNs with pathological confirmation were retrospectively collected from the three medical institutions from January 2019 to December 2021 (Hospital 1), January 2016 to December 2018 (Hospital 2), and January 2020 to June 2022 (Hospital 3). The inclusion criteria were as follows (1): Maximum axial diameter of GGNs on baseline CT between 5mm and 30mm; (2) Baseline thin-slice (≤ 1.5mm) chest CT scans; (3) Surgery was performed within one month after the last scan; (4) For multiple GGNs, only the nodule with the highest risk of malignancy or the largest initial diameter was included. The exclusion criteria were as follows: (1) Preoperative anti-cancer therapy; (2) Loss of clinical information or thin slice image data; (3) Artifacts or any other factors affecting the display of GGNs. All CT images in this study were plain scan images. Our criteria for benign and malignant evaluation were based on the 2021 edition of the World Health Organization classification recommendations (24); therefore, the precursor glandular lesions (i.e., atypical adenomatous hyperplasia, AAH, and adenocarcinoma in situ, AIS) were classified as benign.

**Figure 1:**
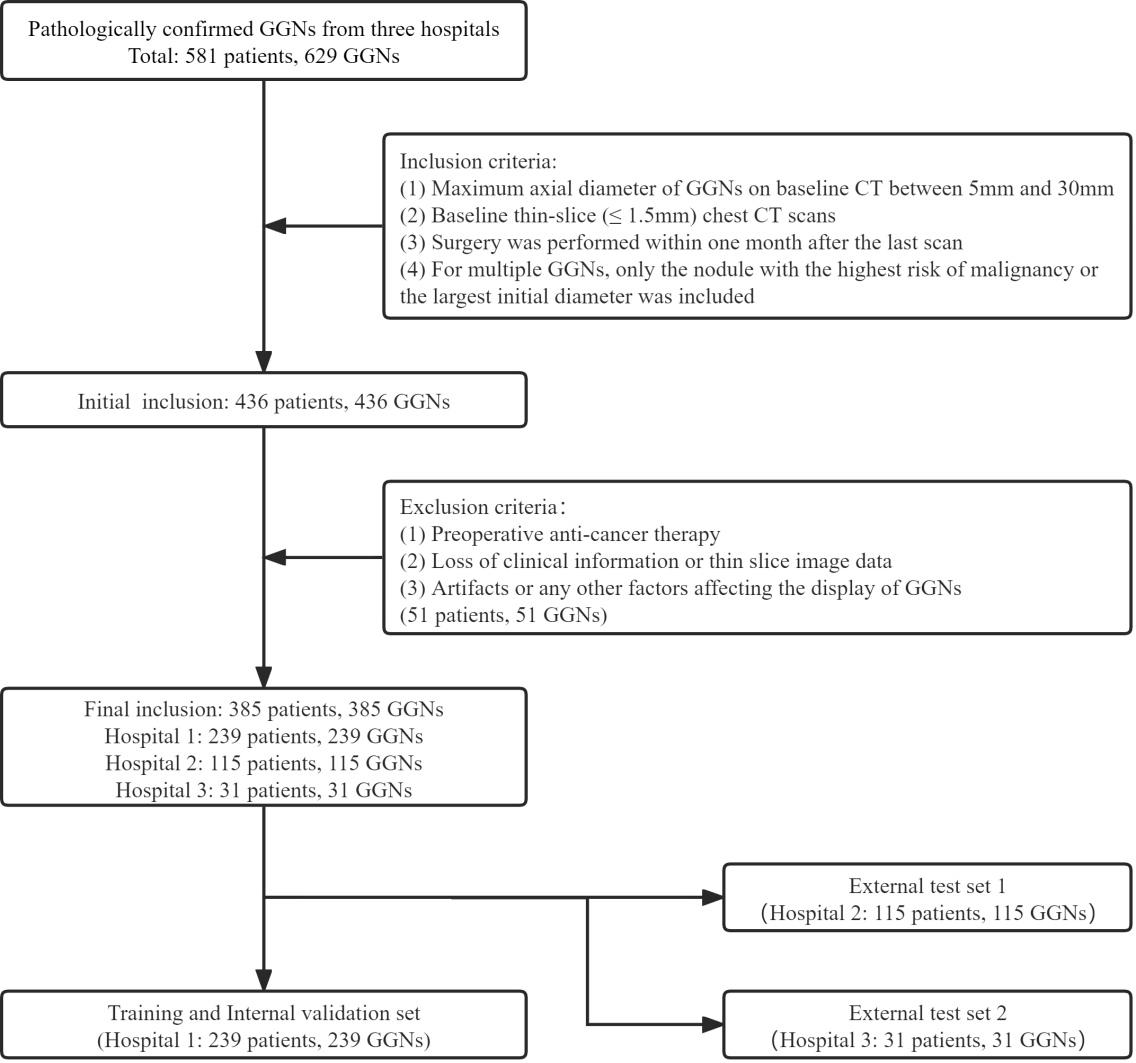
The inclusion and allocation of patients with pathological results.

**Figure 2:**
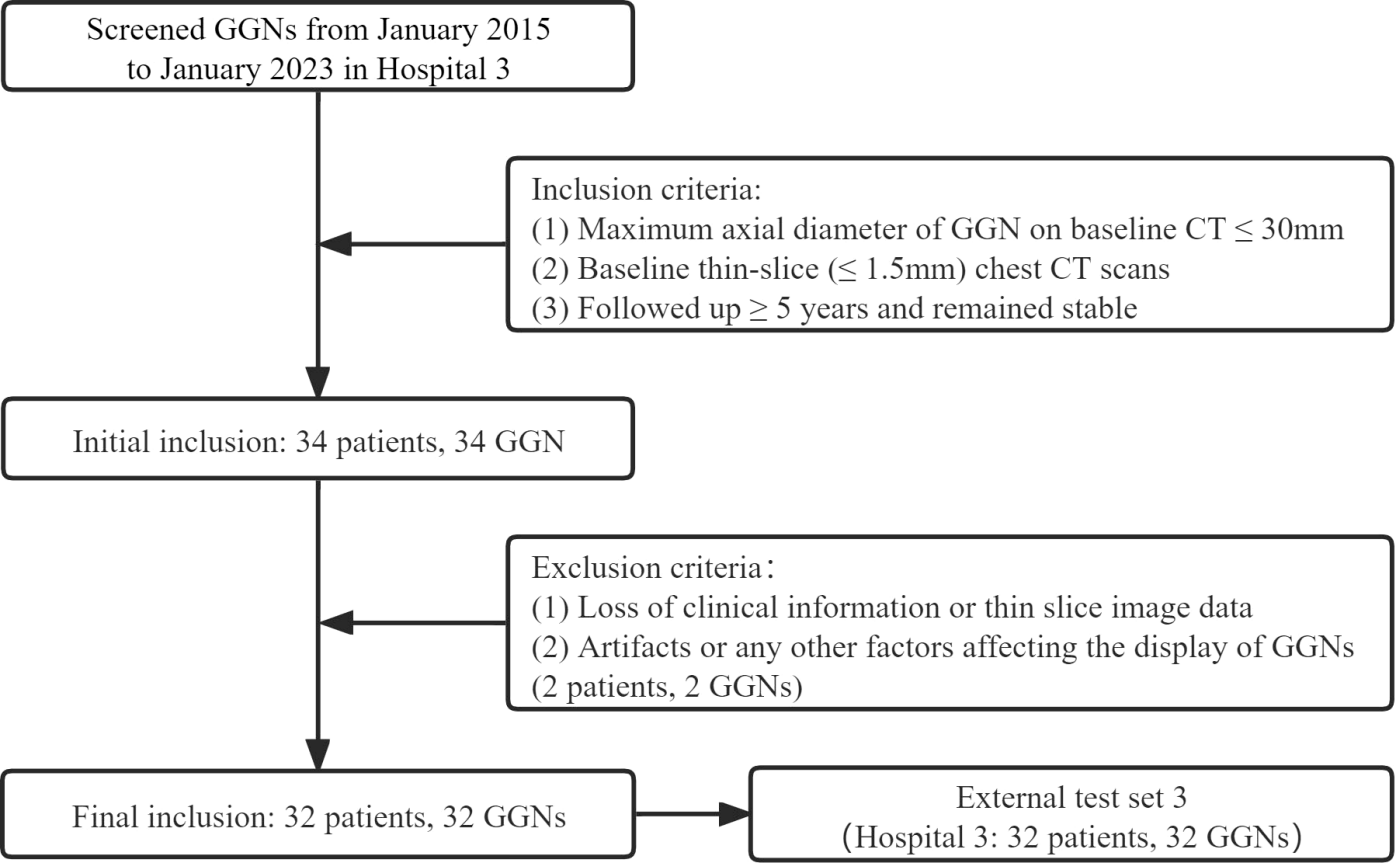
The inclusion and allocation of patients without pathological results.

Finally, 385 GGNs (149 benign and 236 malignant) of 385 patients were included (**Figure 1**). To maximize the training effect, we divided the data of Hospital 1 (239 patients, 239 GGNs) into a training set and an internal validation set at a ratio of 6:4 according to the composition of benign and malignant GGNs. Two independent external test sets were from Hospital 2 (115 patients, 115 GGNs) and Hospital 3 (31 patients, 31 GGNs). In addition, to further verify our model’s generalization, we screened 32 GGNs from Hospital 3 that were followed up over five years and still stable from January 2015 to January 2023 to form an independent external test set 3 (**Figure 2**). None of the GGNs in the external test set 3 had been pathologically confirmed to be benign or malignant, and given their prolonged stable state, they were treated as benign GGNs for analysis. The ethics committee of Hospital 2 approved the study, and the patient’s informed consent was waived because of the study’s retrospective nature.

### 2.2 Image acquisition

All CT images were retrieved from the picture archiving and communication system (PACS) and saved in digital imaging and communications in medicine (DICOM) format. The image acquisition equipment is as follows: GE MEDICAL SYSTEMS Discovery HD750 CT, GE MEDICAL SYSTEMS Optima CT670, Philips Brilliance iCT, Philips Ingenuity CT, Siemens SOMATOM Force and Siemens SOMATOM Sensation 64 (detailed scan and reconstruction parameters are shown in **Table 1**).

**Table 1:** CT scan and reconstruction parameters.

Scanning equipment	GE		Philips		Siemens SOMATOM	
	Discovery HD750	Optima CT670	Brilliance iCT	Ingenuity CT	Force	Sensation 64
Hospital	1	1	2	2	3	3
Tube voltage	120kVp	120kVp	120kVp	120kVp	120kVp	120kVp
Tube current	260mA	200mA	AEC	AEC	200mA	200mA
Pitch	1.375	1.375	0.8	1.02	2.0	0.8
Collimation	0.625mm×64	0.625mm×64	0.625mm×128	0.625mm×128	0.6mm×96	0.75mm×64
Rotation time	0.7s/rot	0.6s/rot	0.5s/rot	0.33s/rot	0.25s/rot	0.35s/rot
SFOV	50cm	50cm	35cm	35cm	50cm	50cm
Slice thickness of reconstruction	1.25mm	1.25mm	0.6/1mm	0.6/1mm	0.6/1mm	1/1.5mm
Slice interval of reconstruction	1.25mm	1.25mm	0.6/1mm	0.6/1mm	0.6/1mm	1/1.5mm
Reconstruction algorithm	STND	STND	LUNG	LUNG	Medium sharp	Medium sharp

AEC: dose modulation with automatic exposure control.

### 2.3 Evaluation of clinical-morphological features

All patients’ clinical information was collected from the electronic medical record system. Four clinical items were collected, including sex, age, smoking status, and family history of lung cancer. All CT morphological features were evaluated with mediastinal window (window width: 400 Hu, window level: -40 HU) and lung window (1400 Hu, -600 HU) settings. Two chest radiologists (WH and JZ, with seven years and 15 years of chest CT diagnostic experience, respectively) were independently evaluated and then checked by another radiologist (LF, with 20 years of chest CT experience). In case of disagreements, a consensus was reached through consultation. All radiologists were blinded to the pathological findings.

CT morphological features included location, size, attenuation, shape, margin, nodule-lung interface, internal features, and adjacent structures. In addition to the lobe in which the nodule was located, we also classified the nodule into three location types based on quantitative definitions of central lung cancer: inner 1/3, middle 1/3, and outer 1/3 (25). The size included the maximum and minimum diameters perpendicular to each other on the axial section. The attenuation was classified into two subtypes according to the presence of solid components or not: pGGNs and mixed ground-glass nodules (mGGNs). The pGGNs were defined as an area of hazy increased lung attenuation with distinct margins of underlying vessels and bronchial walls; the mGGNs were defined as nodules with both ground-glass and solid components. Shapes were classified as irregular or round/oval. Margin features included lobulation, spiculation, and spine-like process. The spine-like process is the structure that extends from the lesion but differs from the boundary of the lung parenchyma by having at least one convex border (26). The nodule-lung interface was classified into three subtypes: ill-defined, well-defined and smooth, and well-defined but coarse (27). The interior features included bubble lucency, cavity, air-containing space, calcification, bronchial cut-off, and distorted/dilated bronchus (26, 27). The adjacent structures included pleural indentation and vascular convergence. In addition, the status of the bronchial wall and emphysema of the whole lung was evaluated.

### 2.4 Whole lung segmentation and radiomics features

Bilateral lung segmentation, separating lung tissue from the chest wall and mediastinum, was automatically carried out with a publicly available 3D deep learning model (23). A manual revision was performed to guarantee accurate segmentation when necessary. The radiomics features were extracted from left, right, and bilateral lung tissues separately with the Pyradiomics library (version 3.0) with the Shukun Medical research platform (Shukun (Beijing) Network Technology Co., Ltd.) (28). All radiomics feature extraction adhered to the Image Biomarker Standardization Initiative (IBSI) recommendations to ensure reproducibility (29). In order to eliminate the variances caused by different scanner acquisitions, the acquired images are preprocessed: normalization, resample to a voxel size of 1×1×1 mm^3^ using B-Spline interpolation and gray-level discretization with a fixed bin width of 25. One hundred seven features extracted from original images consisted of 14 shape-based, 18 first-order statistics features, 24 gray-level cooccurrence matrix features, 14 gray-level dependence matrix features, 16 gray-level run-length matrix features, 16 gray-level size zone matrix features, and 5 neighboring gray-tone difference matrix features. Besides, 14 image filters were applied to the original images, thus yielding derived images based on which additional features were extracted. Finally, a total of 1409 radiomics features were extracted.

### 2.5 Construction of the neural network

#### 2.5.1 Data preprocessing

We cleaned and processed text items (clinical data, morphological features) and whole-lung radiomics features before feeding them into the network. In order to facilitate the input into the network, all text items were replaced by numbers. Then z-score standardization was used to process the whole-lung radiomics features with huge data dispersion to prevent the situation that it was challenging to obtain features or fit because of the large dispersion when entering the network.

#### 2.5.2 The first-order neural network

We used the back-propagation neural network (BPNN) as the first-order network for the DL model based on clinical-morphological features and whole-lung radiomics features. The BPNN consists of a convolutional block and a fully connected block. The first-order BPNN computed correlations between features from the input data and then used the fully connected block network to compute a 2*2*2 matrix based on the computed correlations and Rectified Linear Unit.

The number of network layers was determined according to the complexity of the input data: we used a 25-layer neural network for morphological features with more items and a 5-layer neural network for clinical features with fewer items. For the vast number of whole-lung radiomics features, we used the BPNN to match the features among them. After learning the training set, the appropriate features were selected automatically, and the relationship between features was adjusted. Using BPNN to adjust the relationship between features automatically will facilitate fitting the proper relationship between features.

We designed a convolutional neural network (CNN) with 26 layers as the first-order network of our DL model based on whole-lung images. The CNN comprises eleven convolutional layers, eleven pooling layers, and four fully connected blocks. The seed point algorithm was used to fill the lung to obtain the internal structure of the lung, and then the whole lung image and its internal tissue features were extracted. According to the description of the location of the patient’s nodules in the morphological features, the corresponding side lung sample was selected. Subsequently, the samples were formatted to whole-lung images of 256*256*256 pixels. After all the images were collected, each formatted whole-lung image was input into the CNN. The decoder network of the fully connected block was used to calculate a 2*2*2 matrix based on the features extracted by the encoder and the Sigmoid function.

#### 2.5.3 The second-order neural network

The second-order neural network was still constructed using BPNN, and the 2*2*2 matrices generated by the first-order network were input into it in batches and multiple times. BPNN automatically calculated the correlations and weights in each matrix and outputted a single value in the range [0, 1] to indicate the probability that the nodule is malignant. Then the benign or malignant nodules were judged by comparing the value with the threshold obtained during training. Those with a value above the threshold were classified as malignant, and below were classified as benign. We used cross-entropy as a loss function during model training. Weights were optimized using an Adam optimizer with an initial learning rate 1e^-3^.

#### 2.5.4 Prediction models

**Figure 3:**
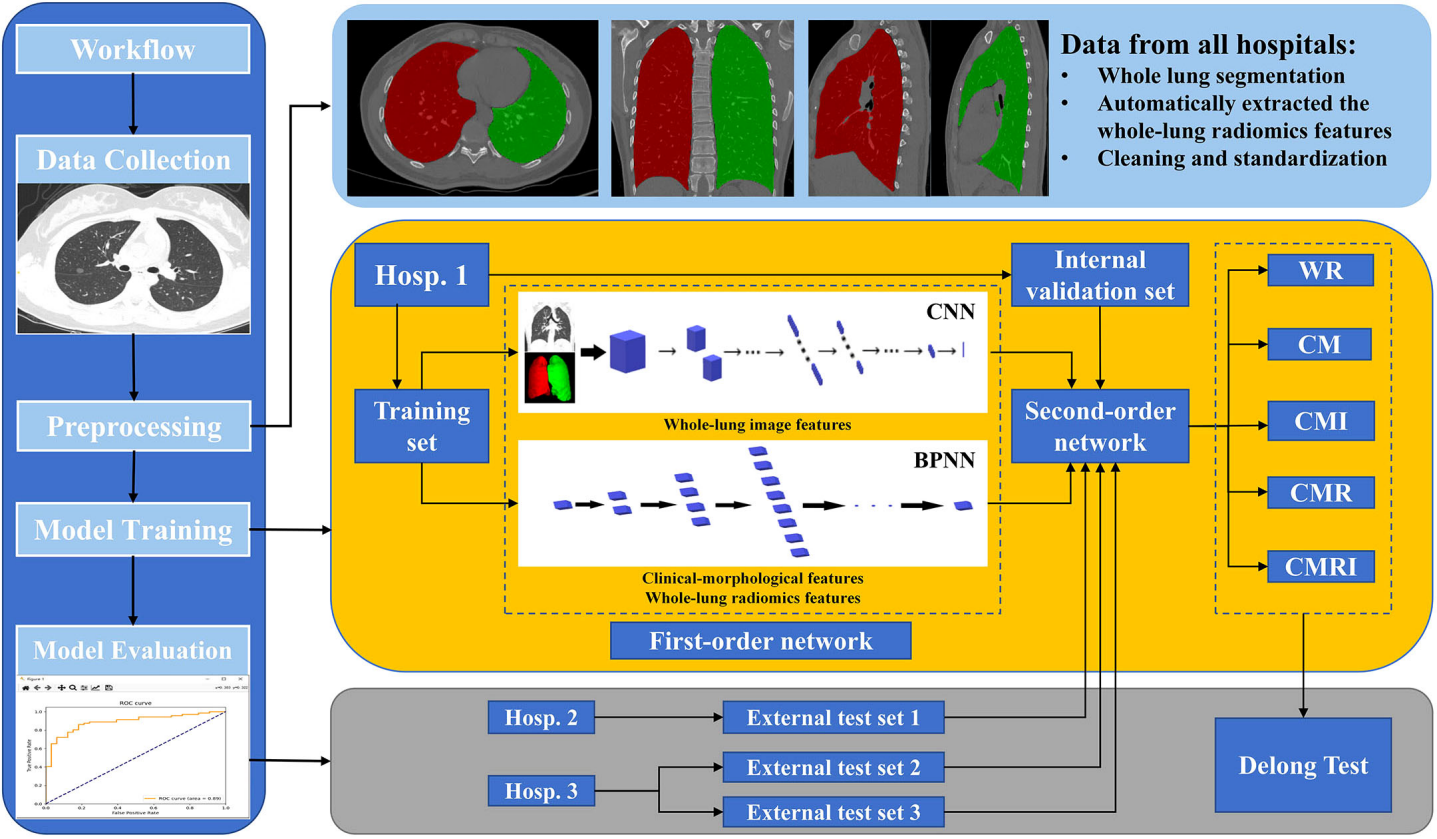
The overall workflow of this study The white square of CNN is the first-order neural network based on the whole-lung image features; the white block of BPNN is the first-order neural network based on clinical-morphological features and whole-lung radiomics features. CNN: convolutional neural network, BPNN: back-propagation neural network, CM: the model based on clinical-morphological features, CMR: the model combined clinical-morphological features and whole-lung radiomics features, CMI: the model combined clinical-morphological features and whole-lung image features, CMRI: the model integrated clinical-morphological features, whole-lung radiomics features, and whole-lung image features, WR: the model based on whole-lung radiomics features.

According to the different data collocations used for the second-order neural network training, we constructed five DL models to predict the benign and malignant GGNs, which are as follows: the model based on clinical-morphological features (CM), the model based on whole-lung radiomics features (WR), the model combined clinical-morphological features and whole-lung radiomics features (CMR), the model combined clinical-morphological features and whole-lung image features (CMI), and the model integrated clinical-morphological features, whole-lung radiomics features, and whole-lung image features (CMRI). The performance of the models was validated in an internal validation set and tested in two external test sets. We plotted the model’s receiver operator characteristic (ROC) curves, calculated the area under the curve (AUC), and compared the difference between AUCs. The overall workflow of this study is presented in **Figure 3**.

### 2.6 Statistical analysis

All statistical analyses were performed using SPSS 23.0 software for Windows (SPSS, Chicago, USA) and Python software (version 3.6.8, Python Software Foundation, USA). The chi-square or Fisher’s exact test was used for qualitative variables, and the Mann-Whitney test was used for quantitative variables. The AUC was used to evaluate the performance of prediction models, and the DeLong test was used to compare the differences in AUC between models pairwise. *P*<0.05 was considered statistically significant.

## 3 Results

### 3.1 Clinical and morphological features of pathologically confirmed GGNs in three hospitals

385 GGNs (243 pGGNs, 142 mGGNs) of 385 patients (268 females, mean age 56.26 ± 11.30 years, range 20-83 years) were collected retrospectively from 3 hospitals. The pathological findings were composed as follows: precursor glandular lesions (N=138, 35.85%), minimally invasive adenocarcinoma (MIA, N=74, 19.22%), invasive adenocarcinoma (IAC, N=161, 41.82%), fibrous or chronic inflammatory nodules (N=6, 1.56%), organizing pneumonia (N=2, 0.52%), tuberculosis (N=1, 0.26%), hamartoma (N=1, 0.26%), pulmonary sclerosing hemangioma (N=1, 0.26%) and squamous cell carcinoma (N=1, 0.26%).

In all three hospitals, compared with benign GGNs, patients with malignant GGNs were older, had larger baseline diameters, and were more likely to show lobulation, spiculation, pleural indentation, and vascular convergence. However, there were no significant differences in sex, family history of lung cancer, and the location of nodules. In addition, some clinical and morphological differences between benign and malignant GGNs were only observed in some hospitals: (1) significant differences in shape and nodule-lung interface were observed in Hospital 1; (2) significant differences in smoking status, bubble lucency, cavity, and air-containing space were observed in Hospital 2; (3) significant differences in emphysema, bronchial wall, spine-like process, bronchial cut-off, and distorted/dilated bronchus were observed in Hospitals 1 and 2 but not in Hospital 3. **Table 2** and **Supplementary Table 1** shows the detail of differences in clinical and morphological features between benign and malignant GGNs in each hospital.

**Table 2:** Clinical information of patients in each set.

Clinical information	Training and internal validation set (Hosp. 1, N=239)			External test set 1 (Hosp. 2, N=115)			External test set 2 (Hosp. 3, N=31)			External test set 3 (Hosp. 3, N=32)
	Benign (n=60)	Malignant (n=179)	*P*	Benign (n=73)	Malignant (n=42)	*P*	Benign (n=16)	Malignant (n=15)	*P*	
Sex Male Female	15 (25.0) 45 (75.0)	52 (29.1) 127(70.1)	0.55	22 (30.1) 51 (69.9)	16 (38.1) 26 (61.9)	0.38	6 (37.5) 10 (62.5)	6 (40.0) 9 (60.0)	1.00*	9 (28.1) 23 (71.9)
Age (Years)	58.00 (12.00)	60.00 (12.00)	**0.04^#^**	50.00 (18.00)	55.00 (12.00)	**0.02^#^**	43.50 (20.00)	55.00 (22.00)	**0.01^#^**	40.00 (23.75)
Smoking status Current/Former Never	9 (15.0) 51 (85.0)	25 (14.0) 154 (86.0)	0.84	7 (9.6) 66 (90.4)	10 (23.8) 32 (76.2)	**0.04**	3 (18.8) 13 (81.3)	4 (26.7) 11 (73.3)	0.69*	7 (21.9) 25 (78.1)
Lung cancer family history Yes No	3 (5.0) 57 (95.0)	5 (2.8) 174 (97.2)	0.42*	1 (1.4) 72 (98.6)	3 (7.1) 39 (92.9)	0.14*	0 (0.0) 16(100.0)	0 (0.0) 15(100.0)	NA	0 (0.0) 32 (100.0)

Age is shown as the median, with the interquartile range in parentheses; other data are shown as the number of patients, with the percentage in parentheses. Fisher exact probability test was used for P values with “*”, Mann-Whitney test was used for those with “#”, and chi-square test was used for those without markers. P values in bold indicate statistical significance. NA, not applicable.

### 3.2 Prediction performance of different models in sets with pathologically confirmed GGNs

**Figure 4:**
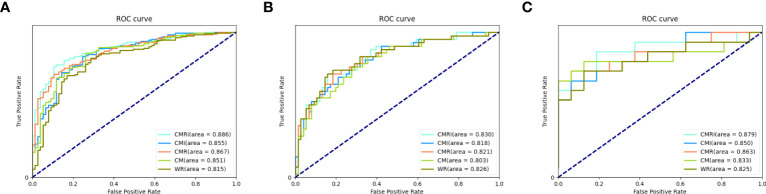
Performance of different models in the prediction of benign and malignant GGN in sets with pathologically confirmed GGNs The ROC curves of five different models in each set are shown in the figure: **(A)** internal validation set, **(B)** external test set 1, and **(C)** external test set 2.

**Figure 5:**
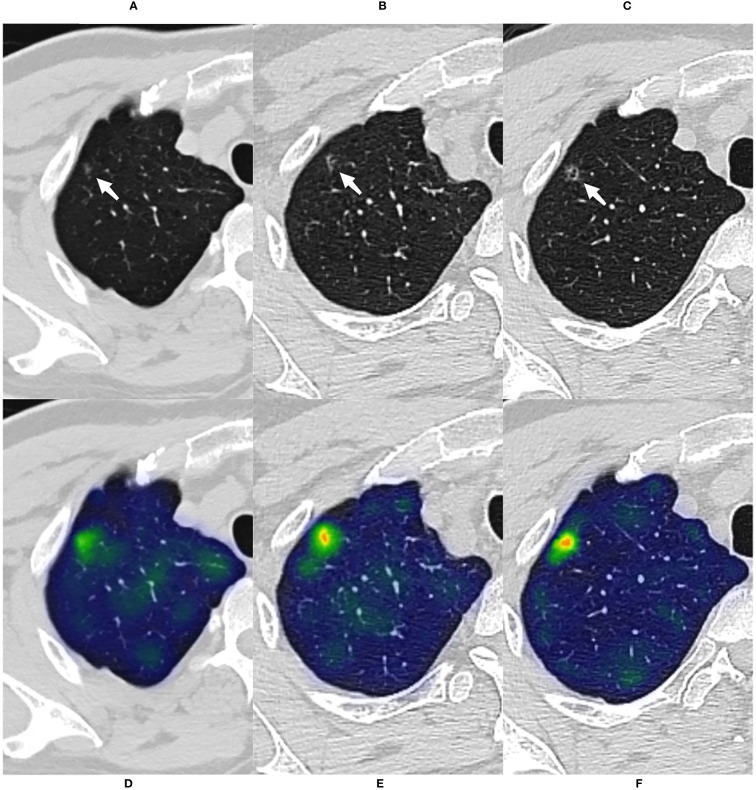
A case of malignant GGN was predicted successfully by the CMRI model The nodule was from the external test set 2. **(A)** A 69-year-old male presented with a small pGGN in the right upper lobe on baseline CT scan(white arrow). **(B)** The first review was performed after 293 days of follow-up and the lesion was slightly enlarged(white arrow). **(C)** A second examination was performed 691 days after follow-up, and the lesion was significantly enlarged and heterogeneous in density(white arrow). Sixteen days after the second review(for a total follow-up of 707 days), the nodule was surgically removed and pathologically confirmed the minimally invasive adenocarcinoma. **(D–F)** Heatmaps generated by GRAD-CAM for baseline, first review, and second review. Red or yellow areas represent high importance or strong activation, while blue or green areas indicate low importance or weak activation. The prediction scores of CM, WR, CMI, CMR and CMRI models were 0.667, 0.670, 0.718, 0.727 and 0.783, respectively. Compare these prediction scores with the threshold (0.764) calculated by the neural network: those with a value above the threshold were classified as malignant, and below were classified as benign. So, The CMRI model predicted this malignant nodule successfully based on the baseline CT features, whereas none of the CM, CMR, CMI, and WR models predicted correctly.

In all three sets, the CMRI model showed the best prediction performance, with an AUC of 0.886 (95% confidence interval[CI]: 0.841~0.921) in the internal validation set (Hospital 1), 0.830 (95% CI: 0.749~0.893) in the external test set 1 (Hospital 2), and 0.879 (95% CI: 0.712~0.968) in the external test set 2 (Hospital 3). WR model performed slightly worse than the other models in the internal validation set (AUC=0.815) and the external test set 2 (AUC=0.825). The CM model performed marginally worse in the external test set 1 (AUC=0.803). **Figure 4** and **Table 3** show the details. In addition, we present a malignant GGN in **Figure 5** predicted by the CMRI model successfully based on baseline CT but failed by the other models.

**Table 3:** Prediction performance of different models in sets with pathologically confirmed GGNs.

Sets	Model	AUC (95% CI)	Accuracy	Sensitivity	Specificity	PPV	NPV
Internal validation Set (Hosp. 1)	CM	0.851 (0.803-0.892)	81.7%	67.5%	87.4%	68.4%	87.0%
	WR	0.815 (0.763-0.859)	78.7%	62.3%	85.3%	84.0%	69.8%
	CMR	0.867 (0.821-0.905)	78.7%	62.3%	85.3%	63.2%	84.9%
	CMI	0.855 (0.807-0.894)	81.0%	66.2%	86.9%	67.1%	86.5%
	CMRI	0.886 (0.841-0.921)	82.5%	68.8%	88.0%	69.7%	87.5%
External test set 1 (Hosp. 2)	CM	0.803 (0.719-0.870)	75.4%	80.3%	66.7%	81.3%	65.1%
	WR	0.826 (0.746-0.890)	70.3%	76.3%	59.5%	63.2%	84.9%
	CMR	0.821 (0.746-0.890)	73.7%	78.9%	64.3%	80.0%	62.8%
	CMI	0.818 (0.737-0.883)	78.8%	82.9%	71.4%	84.0%	69.8%
	CMRI	0.830 (0.749-0.893)	77.1%	81.6%	69.0%	82.7%	67.4%
External test set 2 (Hosp. 3)	CM	0.833 (0.656-0.942)	77.4%	75.0%	80.0%	80.0%	75.0%
	WR	0.825 (0.647-0.937)	70.9%	68.8%	73.3%	73.3%	68.8%
	CMR	0.863 (0.691-0.959)	83.9%	81.3%	86.7%	86.7%	81.3%
	CMI	0.850 (0.676-0.952)	71.0%	75.0%	73.3%	73.4%	68.8%
	CMRI	0.879 (0.712-0.968)	83.9%	75.0%	80.0%	86.7%	81.3%

AUC, area under the receiver operator characteristic curve; 95%CI, 95% confidence interval; PPV, positive predictive value; NPV, negative predictive value.

### 3.3 Pairwise comparison of AUC between five models in sets with pathologically confirmed GGNs

In the internal validation set, the differences in AUC between all five models were significant. In the external test set 1, there was no significant difference in AUC between the CMI and the CMR models (*P*=0.1048), and the AUC differences between the other models were statistically significant. In the external test set 2, there was no significant difference in AUC between the CMI and the WR models (*P*=0.1092), and the AUC differences between the other models were statistically significant. **Table 4** shows the details.

**Table 4:** Pairwise comparison of AUC between five models in sets with pathologically confirmed GGNs.

Database	Model	CM	WR	CMR	CMI	CMRI
Internal validation Set (Hosp. 1)	CM	NA	–	–	–	–
	WR	0.0373	NA	–	–	–
	CMR	0.0144	0.0374	NA	–	–
	CMI	0.0423	0.0478	0.0269	NA	–
	CMRI	0.0181	0.0001	0.0245	0.0153	NA
External test set 1 (Hosp. 2)	CM	NA	–	–	–	–
	WR	0.0071	NA	–	–	–
	CMR	0.0030	0.0285	NA	–	–
	CMI	0.0009	0.0416	**0.1048**	NA	–
	CMRI	0.0085	0.0208	0.0365	0.0245	NA
External test set 2 (Hosp. 3)	CM	NA	–	–	–	–
	WR	0.0152	NA	–	–	–
	CMR	0.0372	0.0196	NA	–	–
	CMI	0.0325	**0.1092**	0.0132	NA	–
	CMRI	0.0099	0.0226	0.0142	0.0116	NA

The data in the table are the P values of the difference in AUC between the models, and the data with no significant difference are shown in bold. AUC, area under the receiver operator characteristic curve; NA, not applicable.

### 3.4 Predictive performance of stable GGNs with long-term follow-up

A total of 32 GGNs (32 patients, 23 females, median age 40 years, range: 24-68 years) with follow-up over five years and remaining stable were collected as the external test set 3. The median follow-up time was 2175 days (range 1855-2895 days). The axial section’s median maximum and minimum diameters were 5.1 mm and 3.8 mm, respectively. **Tables 2** and **3** show the detailed clinical and morphological features.

**Figure 6:**
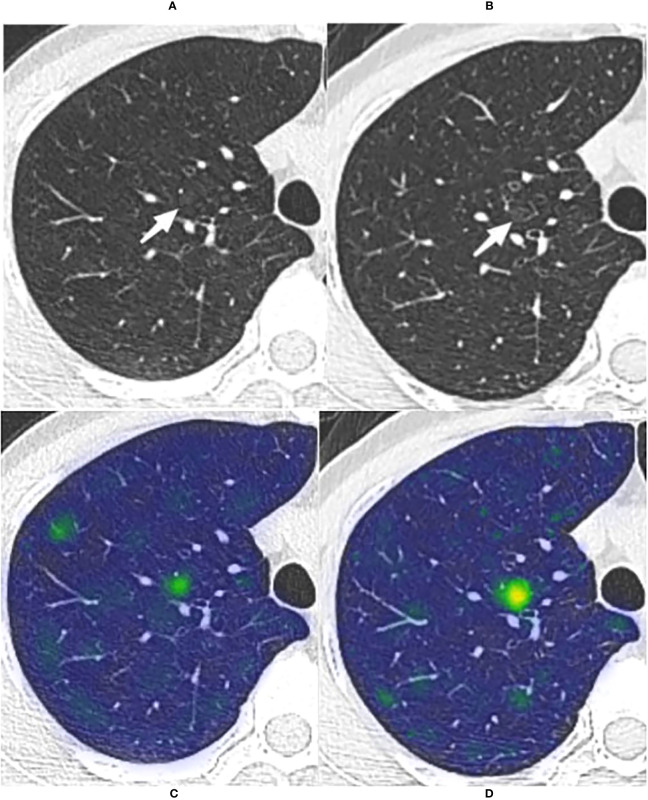
The long-term stable GGN that incorrectly predicted by the WR model The nodule was from the external test set 3 (without pathologically confirmed, all considered benign GGNs). **(A, B)** Are chest CT images of a 45-year-old female with a slice thickness of 1.5mm and 1.25mm, respectively. **(A)** Baseline CT showed a faint pGGN (white arrow) in the right upper lobe. **(B)** Follow-up CT of 2609 days (7.1 years) after baseline showed that the nodule was stable. This nodule was correctly predicted by four models other than the WR model. **(C, D)** Show the baseline and the follow-up heatmaps generated by GRAD-CAM, respectively. The prediction scores of CM, WR, CMI, CMR and CMRI models were 0.114, 0.799, 0.082, 0.103 and 0.094, respectively. Only the WR model had a prediction score above the threshold (0.764); therefore, This nodule was correctly predicted by four models other than the WR model. Although the nodule has not changed significantly after 7.1 years of follow-up, the heatmaps **(D)** activity is still increased compared with that of **(C)**, which may indicate its slow progression.

Since all 32 GGNs were considered benign cases, and malignant cases used for comparison were lacking, we only evaluated the accuracy of the prediction results of the model. The prediction accuracy of the five models was 100% (32/32, CM), 93.75% (30/32, WR), 96.88% (31/32, CMI), 96.88% (31/32, CMR), and 96.88% (31/32, CMRI), respectively. The CMI, CMR, and CMRI models incorrectly predicted the same nodule. The WR model incorrectly predicted two nodules, one of which was the same nodule as the other models incorrectly predicted. We showed in **Figure 6** the CT images of the initial and the most recent follow-up of the nodule that only the WR model incorrectly predicted.

## 4 Discussion

Lung cancer remains the leading cause of cancer death globally (30). The high malignant probability of GGNs necessitates detailed management recommendations (31, 32). At the same time, the slow growth and atypical morphological characteristics of GGNs also make the differentiation between benign and malignant GGNs more challenging (33–35). Currently, most artificial intelligence (AI) models for predicting benign and malignant pulmonary nodules were built based on the nodules’ local features or combined with the feature within a specific range around the nodule. Unlike these studies, we chose the DL model based on whole-lung CT features (radiomics and image features) for benign and malignant prediction of GGNs.

Our results showed that the WR model, based on whole-lung radiomics features, could predict benign and malignant GGNs, and the AUC in three sets was 0.815, 0.826, and 0.825, respectively. However, the WR model showed no significant advantage over the other models, and the CM model even performed slightly better in the internal validation (AUC=0.851) and the external test set 1 (AUC=0.833). Furthermore, the CMR and CMI models performed better than the CM models in three pathological confirmed sets, respectively. Previous studies (36, 37) have shown that the presence of diseases such as emphysema and fibrosis are generally associated with poor prognosis and are considered precancerous diseases. These precancerous diseases often involve a more extensive range of lung parenchyma than lung nodules. Therefore, the features of relevant pathological regions may be helpful information in the massive lung features to make the benign and malignant prediction of GGNs and further improve the prediction performance.

The CMRI model combining all features achieved the best AUC in the three sets, with an improvement of 7.1% (internal validation set), 2.7% (external test set 1), and 5.4% (external test set 2) compared to the lowest AUC model in each set, respectively. The results of the Delong test showed that the AUC of the CMRI model in three sets was significantly different from those of other models in the same set, further indicating that the whole-lung features indeed improved the discrimination ability of the models. Masquelin et al. proposed a standardized method for extracting features around nodules based on secondary pulmonary lobules (14). The performance of the malignant tumor prediction model that integrated nodules and surrounding lung parenchyma features (within the range of 10 mm or 15 mm) was higher than that of using nodule features or surrounding features alone. The improvement in prediction performance was also independent of the type of machine learning algorithm. The range of whole-lung features we extracted included the secondary lobules, which obtained similar good predictive performance.

Moreover, one study proposed a DL-based local-global model (including nodule and whole-lung information) to differentiate nodular cryptococcosis from lung cancer (16). The effect (AUC=0.88) of the local-global model was better than the model only based on the nodule’s features (AUC=0.84). Another study found that image features mined from the whole lung were related to multiple critical gene pathways related to drug resistance or cancer progression mechanisms, which could provide additional prognostic information for targeted lung cancer therapy (17). All these studies have shown the additional diagnostic and predictive value of a broader range of lung parenchymal features for local lesions.

The clinical and morphological features of the GGNs help distinguish benign from malignant nodules. Patients with malignant GGNs were older in all three hospitals, mostly mGGNs, and had larger initial diameters. Previous studies have shown that age and larger diameter are risk factors for malignant GGN growth, consistent with our results (38–41). The appearance of MIA on CT images is usually mGGNs, and the solid component indicates the extent of tumor invasion (38). In this study, the proportion of malignant lesions in mGGNs was 93.75%, 64.71%, and 76.92% in three hospitals, respectively, confirming that mGGNs were more likely to be malignant. Lobulation, spiculation, pleural indentation, and vascular convergence occurred more frequently in malignant GGNs from three hospitals, consistent with the previous studies (26, 27).

However, some clinical and morphological differences between benign and malignant GGNs were inconsistent in the three hospitals. For example, smoking and family history are recognized risk factors for lung cancer, but we observed this difference only in Hospital 3. Female is closely related to lung cancer (42), but there is no significant sex difference between benign and malignant GGNs in the three hospitals. Some features were significantly different only in Hospitals 1 and 2 but not in Hospital 3. The following reasons may be relevant: 1) the origin of cases in all hospitals is different, and there is a selection bias; 2) the proportion of benign GGNs in Hospital 2 (63.5%, external test set 1) was significantly higher than that in the other two hospitals (25.1% and 51.6%); 3) the number of GGNs (N=31) in Hospital 3 (external test set 2) is less. The higher proportion of benign GGNs may explain why the AUC and accuracy of the model in the external test set 1 were weaker than those in the other two sets. In addition, previous studies (43, 44) have shown conflicting results on the relationship between the interface of GGNs and malignancy, with both well- and ill-defined interfaces appearing to be significantly associated with malignant GGNs. In the present study, we only observed a higher frequency of well-defined but coarse interfaces in malignant GGNs from Hospital 1. In addition to the above reasons, differences in the observer’s subjective evaluations of interfaces are also related. Furthermore, subjective differences also show the limitation of differentiating benign and malignant GGNs based on morphological features. Hence, a more extensive and balanced database of nodules is the key to improving the model.

The results of the Delong test showed that the differences in AUC between CMI and CMR models in the external test set 1 and between CMI and WR models in the external test set 2 were not statistically significant. The fact that the AUCs of the CMI (AUC=0.818) and CMR (AUC=0.821) models were too close may be the reason for the non-significant difference. In the external test set 2, although the AUC of the CMI model was 2.5% higher than that of the WR model, this may occur by chance due to the small sample size of this set and the increased weight of individual data on the influence of the model.

In the current study, we used 32 long-term (≥ 5 years) stable GGNs without pathological confirmation for models’ further validation. Fleischner Society recommends no routine follow-up for subsolid nodules with a size < 6mm (4). Even in subsolid nodules ≥ 6mm, the growth rate after five years of stabilization is only 2%, and the growth of these nodules has no clinical effect (45). So we considered these GGNs benign based on their long-term stable state and smaller initial size (median maximum diameter, 5.1mm). Surprisingly, the CM model achieved 100% accuracy, while the other four models based on whole-lung radiomics features and whole-lung image features showed prediction accuracies ranging from 93.75% to 98.68%. The high accuracy of the CM model may be related to the smaller size and fewer positive CT morphologic features. Errors in the other four models may be the combined effects of incorporating whole-lung features. In addition, there may be an overestimation of the accuracy exhibited by all models due to the lack of pathological results. Some studies have found that the indolent growth nature of GGNs determines that the final pathological result may still be malignant even after maintaining long-term stability (33, 34, 46). The WR model (only based on whole-lung radiomics features) incorrectly predicted without combining clinical and morphological features may also suggest the presence of information in the whole-lung features to differentiate the entirely benign or malignant of this type of GGNs. Lee et al. also found that 13% (27/208) of subsolid nodules grew after five years of stability, and about 95% of these nodules were less than 6mm in size (47). Therefore, despite the bias in the external test set 3 results, it further illustrates the feasibility of the method for predicting benign and malignant GGNs based on whole-lung features and the generalization potential of the DL model.

Technically, we solved the following problems. The first is to design a second-order neural network to effectively integrate clinical-morphological, image, and radiomics features. The second-order neural network better fitted the relationship between the three features and restored their genuine connection as much as possible. Second, we encountered the problem of fewer samples during training. We used the data enhancement method, similar to some previous studies (44, 48), in which samples were shifted and rotated to increase the diversity of samples, improve the accuracy and generalization ability of the model, reduce over-fitting, and improve the accuracy and robustness of the model.

Our study has several limitations. First, the present study was retrospective. Most GGNs (92.3%, 385/417) were confirmed by postoperative pathology. These nodules were already biased toward a malignant probability diagnosis, and final benign GGNs are less than malignant ones, so selection bias was inevitable. Second, our model did not compare with the model based on the local features of the nodule. It is still being determined whether the model based on whole-lung features has an advantage over the model based on local features of the nodule, and further research is needed. Third, other smaller GGNs may exist in the ipsilateral lung where the target GGN is located, and the features of such small GGNs may affect the predictive performance of the model. Finally, a common issue for AI is that the features that entered the model for the differentiation of benign and malignant GGNs needed to be clarified due to the complexity and multidimensionality of DL. Fortunately, we have shown that whole-lung features can be used to predict benign and malignant GGNs.

In conclusion, predicting the benign and malignant GGNs by the features extracted from the whole lung is feasible. The CMRI model that integrated clinical-morphological features, whole-lung radiomics features, and whole-lung image features had the best classification performance. The DL model based on whole-lung CT features can provide non-invasive and low-cost prediction and save the time of nodule segmentation. In addition, using the whole lung information to explore the local lesions is helpful to supplement new information beyond the characteristics of the nodules themselves. At the same time, there is also the possibility of multi-task collaboration with other situations that need to be applied to the whole-lung CT features, which is helpful for the more scientific and satisfactory management of GGNs.

## Data availability statement

The original contributions presented in the study are included in the article/**Supplementary Material**. Further inquiries can be directed to the corresponding author.

## Ethics statement

The studies involving humans were approved by The ethics committee of Changzheng Hospital. The studies were conducted in accordance with the local legislation and institutional requirements. The ethics committee/institutional review board waived the requirement of written informed consent for participation from the participants or the participants’ legal guardians/next of kin because the study’s retrospective nature.

## Author contributions

WH: Conceptualization, investigation, methodology, formal analysis, validation, visualization, writing − original draft, writing − review and editing. HD: Conceptualization, Methodology, software, formal analysis, validation, visualization, writing − original draft, writing − review and editing. ZL: Conceptualization, formal analysis, methodology, data curation, validation, visualization, writing − original draft, writing − review and editing. ZX: Methodology, software, validation, visualization, writing − review & editing. TZ: investigation, data curation, formal analysis, writing − review & editing. YMG: investigation, data curation, formal analysis, writing − review and editing. JZ: Investigation, data curation, writing − review & editing. WJ: investigation, data curation, writing − review and editing. YYG: Methodology, software, writing − review and editing. XW: Investigation, data curation, writing − review & editing. WT: formal analysis, funding acquisition, writing − review and editing. PD: Conceptualization, Methodology, investigation, resources, supervision, writing − review and editing. SL: Conceptualization, funding acquisition, methodology, project administration, resources, supervision, writing − review and editing. LF: Conceptualization, funding acquisition, methodology, project administration, resources, supervision, writing − review and editing. All authors contributed to the article and approved the submitted version.
