# Supplementary material for: Baseline whole-lung CT features deriving from deep learning and radiomics: prediction of benign and malignant pulmonary ground-glass nodules
Source: Front Oncol. 2023 Aug 17;13:1255007. doi: 10.3389/fonc.2023.1255007 (PMC10470826; doi:10.3389/fonc.2023.1255007)
Supplement: Supplementary file 1 [file Table_1.docx]

**Supplementary Table 1 Morphological features of nodules in each set**

| Morphological features |  | Training and internal validation set  (Hosp. 1, N=239) | | |  | External test set 1  (Hosp. 2, N=115) | | |  | External test set 2  (Hosp. 3, N=31) | | |  | External test set 3  (Hosp. 3, N=32) |
| --- | --- | --- | --- | --- | --- | --- | --- | --- | --- | --- | --- | --- | --- | --- |
|  |  | Benign  (n=60) | Malignant  (n=179) | *P*  value |  | Benign  (n=73) | Malignant  (n=42) | *P*  value |  | Benign  (n=16) | Malignant  (n = 15) | *P*  value |  |  |
| Subtype  pGGN  mGGN |  | 53 (88.3)  7 (11.7) | 74 (41.3)  105 (58.7) | **0.00** |  | 67 (91.8)  6 (8.2) | 31 (73.8)  11 (26.2) | **0.01** |  | 13 (81.3)  3 (18.8) | 5 (33.3)  10 (66.7) | **0.01*** |  | 26 (81.2)  6 (18.8) |
| Location (concentric lines arising from the hilum & concentric lines from the midline)  Inner one-third  Middle one-third  Outer one-third |  | 1 (1.7)  19 (31.7)  40 (66.7) | 14 (7.8)  64 (35.8)  101 (56.4) | 0.16 |  | 5 (6.8)  28 (38.4)  40 (54.8) | 3 (7.1)  10 (23.8)  29 (69.0) | 0.27 |  | 1 (6.3)  7 (43.8)  8 (50.0) | 1 (6.7)  7 (46.7)  7 (46.7) | 1.00* |  | 1 (3.1)  12 (37.5)  19 (59.4) |
| Location (lobe)  RUL  RML  RLL  LUL  LLL |  | 28 (46.7)  5 (8.3)  12 (20.0)  7 (11.7)  8 (13.3) | 61 (34.1)  14 (7.8)  30 (16.8)  40 (22.3)  34 (19.0) | 0.23 |  | 35 (47.9)  4 (5.5)  11 (15.1)  15 (20.5)  8 (11.0) | 14 (33.3)  3 (7.1)  10 (23.8)  8 (19.0)  7 (16.7) | 0.51 |  | 4 (25.0)  2 (12.5)  1 (6.3)  6 (37.5)  3 (18.8) | 8 (53.3)  1 (6.7)  1 (6.7)  3 (20.0)  2 (13.3) | 0.63* |  | 7 (21.9)  2 (6.2)  7 (21.9)  7 (21.9)  9 (28.1) |
| Emphysema (The lobe with the nodule)  Present  Absent |  | 8 (13.3)  52 (86.7) | 59 (33.0)  120 (67.0) | **0.00** |  | 6 (8.2)  67 (91.8) | 10 (23.8)  32 (76.2) | **0.02** |  | 3 (18.8)  13 (81.3) | 4 (26.7)  11 (73.3) | 0.69* |  | 0 (0.0)  32 (100.0) |

| **Supplementary Table 1 Morphological features of nodules in each set** | | | | | | | | | | | | | | |
| --- | --- | --- | --- | --- | --- | --- | --- | --- | --- | --- | --- | --- | --- | --- |
| Morphological features |  | Training and internal validation set  (Hosp. 1, N=239) | | |  | External test set 1  (Hosp. 2, N=115) | | |  | External test set 2  (Hosp. 3, N=31) | | |  | External test set 3  (Hosp. 3, N=32) |
|  |  | Benign  (n=60) | Malignant  (n=179) | *P*  value |  | Benign  (n=73) | Malignant  (n=42) | *P*  value |  | Benign  (n=16) | Malignant  (n = 15) | *P*  value |  |  |
| Emphysema (Other lobes)  Present  Absent |  | 16 (26.6)  44 (73.3) | 69 (38.5)  110 (61.5) | 0.10 |  | 9 (12.3)  64 (87.7) | 13 (31.0)  29 (69.0) | **0.01** |  | 5 (31.3)  11 (68.8) | 5 (33.3)  10 (66.7) | 1.00* |  | 1 (3.1)  31 (96.9) |
| Bronchial wall  Thickening  Normal |  | 12 (20.0)  48 (80.0) | 78 (43.6)  101 (56.4) | **0.00** |  | 4 (5.5)  69 (94.5) | 9 (21.4)  33 (78.6) | **0.01*** |  | 2 (12.5)  14 (87.5) | 3 (20.0)  12 (80.0) | 0.65* |  | 0 (0.0)  32 (100.0) |
| Size (axial section, mm)  Maximum diameter  [Minimum](javascript:;) diameter |  | 11.05 (5.0)  8.35 (3.6) | 18.20 (10.4)  12.80 (6.9) | **0.00^#^**  **0.00^#^** |  | 10.30 (2.50)  8.30 (2.50) | 13.60 (5.20)  10.40 (4.10) | **0.00^#^**  **0.00^#^** |  | 7.50 (2.50)  6.10 (1.90) | 11.90 (6.80)  8.10 (7.20) | **0.01^#^**  **0.02^#^** |  | 5.10 (1.48)  3.80 (1.18) |
| Shape  Round or Oval  Irregular |  | 56 (93.3)  4 (6.7) | 135 (75.4)  44 (24.6) | **0.00** |  | 71 (97.3)  2 (2.3) | 36 (85.7)  6 (14.3) | 0.05* |  | 16 (100.0)  0 (0.0) | 3 (20.0)  12 (80.0) | 0.10* |  | 31 (96.9)  1 (3.1) |
| Lobulation  Present  Absent |  | 19 (31.7)  41 (68.3) | 129 (72.1)  50 (27.9) | **0.00** |  | 2 (2.3)  71 (97.3) | 20 (47.6)  22 (52.4) | **0.00** |  | 2 (12.5)  14 (87.5) | 12 (80.0)  3 (20.0) | **0.00*** |  | 2 (6.2)  30 (93.8) |
| [Spiculation sign](javascript:;)  Present  Absent |  | 4 (6.7)  56 (93.3) | 78 (43.6)  101 (56.4) | **0.00** |  | 2 (2.3)  71 (97.3) | 8 (19.0)  34 (81.0) | **0.01*** |  | 2 (12.5)  14 (87.5) | 9 (60.0)  6 (40.0) | **0.01*** |  | 0 (0.0)  32 (100.0) |
| Spine-like process  Present  Absent |  | 22 (36.7)  38 (63.3) | 140 (78.2)  39 (21.8) | **0.00** |  | 1 (1.4)  72 (98.6) | 10 (23.8)  32 (76.2) | **0.00*** |  | 1 (6.3)  15 (93.8) | 5 (33.3)  10 (66.7) | 0.08* |  | 1 (3.1)  31 (96.9) |
| Interface  Ill-defined  Well-defined and smooth  Well-defined but coarse |  | 7 (11.7)  2 (3.3)  51 (85.0) | 9 (5.0)  0 (0.0)  170 (95.0) | **0.01*** |  | 6 (8.2)  17 (23.3)  50 (68.5) | 1 (2.4)  5 (11.9)  36 (85.7) | 0.15* |  | 4 (25.0)  3 (18.8)  9 (56.2) | 2 (13.3)  5 (33.3)  8 (53.4) | 0.64* |  | 0 (0.0)  16 (50.0)  16 (50.0) |
| Bubble lucency  Present  Absent |  | 5 (8.3)  55 (91.7) | 28 (15.6)  151 (84.4) | 0.16 |  | 6 (8.2)  67 (91.8) | 10 (23.8)  32 (76.2) | **0.02** |  | 0 (0.0)  16 (100.0) | 1 (6.7)  14 (93.3) | 0.48* |  | 0 (0.0)  32 (100.0) |
| [Cavity](javascript:;)  Present  Absent |  | 1 (1.7)  59 (98.3) | 0 (0.0)  179 (100.0) | 0.25* |  | 0 (0.0)  73 (100.0) | 3 (7.1)  39 (92.9) | **0.04*** |  | 0 (0.0)  16 (100.0) | 0 (0.0)  15 (100.0) | NA |  | 0 (0.0)  32 (100.0) |
| Air containing space  Present  Absent |  | 2 (3.3)  58 (96.7) | 11 (6.1)  168 (93.9) | 0.53* |  | 1 (1.4)  72 (98.6) | 7 (16.7)  35 (83.3) | **0.00*** |  | 1 (6.3)  15 (93.8) | 2 (13.3)  13 (86.7) | 0.60* |  | 0 (0.0)  32 (100.0) |
| [Calcification](javascript:;)  Present  Absent |  | 0 (0.0)  60 (100.0) | 1 (0.6)  178 (96.4) | 1.00* |  | 0 (0.0)  73 (100.0) | 0 (0.0)  42 (100.0) | NA |  | 0 (0.0)  16 (100.0) | 0 (0.0)  15 (100.0) | NA |  | 0 (0.0)  32 (100.0) |
| Bronchial cut-off  Present  Absent |  | 0 (0.0)  60 (100.0) | 47 (26.3)  132 (73.7) | **0.00*** |  | 0 (0.0)  73 (100.0) | 4 (9.5)  38 (90.5) | **0.02*** |  | 0 (0.0)  16 (100.0) | 2 (13.3)  13 (86.7) | 0.23* |  | 0 (0.0)  32 (100.0) |
| Distorted/Dilated bronchus  Present  Absent |  | 8 (13.3)  52 (86.7) | 66 (36.9)  113 (63.1) | **0.00** |  | 10 (13.7)  63 (86.3) | 17 (40.5)  25 (59.5) | **0.00** |  | 2 (12.5)  14 (87.5) | 6 (40.0)  9 (60.0) | 0.11* |  | 0 (0.0)  32 (100.0) |
| Pleural retraction  Present  Absent |  | 11 (18.3)  49 (81.7) | 98 (54.7)  81 (45.3) | **0.00** |  | 2 (2.3)  71 (97.3) | 12 (28.6)  30 (71.4) | **0.00** |  | 0 (0.0)  16 (100.0) | 7 (46.7)  8 (63.3) | **0.00*** |  | 0 (0.0)  32 (100.0) |
| Vessel convergence  Present  Absent |  | 1 (1.7)  59 (98.3) | 51 (28.5)  128 (71.5) | **0.00** |  | 0 (0.0)  73 (100.0) | 5 (11.9)  37 (88.1) | **0.01*** |  | 0 (0.0)  16 (100.0) | 4 (26.7)  11 (73.3) | **0.04*** |  | 0 (0.0)  32 (100.0) |

Size is shown as the median, with the interquartile range in parentheses; other data are shown as the number of nodules, with the percentage in parentheses. Fisher exact probability test was used for P values with "*", Mann-Whitney test was used for those with "^#^", and chi-square test was used for those without markers. *P* values in bold indicate statistical significance. pGGN = pure ground-glass nodule, mGGN = mixed ground-glass nodule, RUL = right upper lobe, RML = right middle lobe, RLL = right lower lobe, LUL = left upper lobe, LLL = left lower lobe, NA = not applicable.
